# RRP9 promotes gemcitabine resistance in pancreatic cancer via activating AKT signaling pathway

**DOI:** 10.1186/s12964-022-00974-5

**Published:** 2022-11-24

**Authors:** Zhiqi Zhang, Haitao Yu, Wenyan Yao, Na Zhu, Ran Miao, Zhiquan Liu, Xuwei Song, Chunhua Xue, Cheng Cai, Ming Cheng, Ke Lin, Dachuan Qi

**Affiliations:** 1 Department of Hepatic-Biliary-Pancreatic Surgery, Shanghai Fourth People’s Hospital, School of Medicine, Tongji University, No.1279 Sanmen Road, Hongkou District, Shanghai, 200434 China; 2 Intensive Care Unit, Qingdao Municipal Hospital, Qingdao, 266001 Shandong Province China; 3 Intensive Care Unit, University-Town Hospital of Chongqing Medical University, Chongqing, 401331 China

**Keywords:** RRP9, IGF2BP1, AKT, Apoptosis, DNA damage, Gemcitabine resistance

## Abstract

**Background:**

Pancreatic cancer (PC) is a highly lethal malignancy regarding digestive system, which is the fourth leading factor of cancer-related mortalities in the globe. Prognosis is poor due to diagnosis at advanced disease stage, low rates of surgical resection, and resistance to traditional radiotherapy and chemotherapy. In order to develop novel therapeutic strategies, further elucidation of the molecular mechanisms underlying PC chemoresistance is required. Ribosomal RNA biogenesis has been implicated in tumorigenesis. Small nucleolar RNAs (snoRNAs) is responsible for post-transcriptional modifications of ribosomal RNAs during biogenesis, which have been identified as potential markers of various cancers. Here, we investigate the U3 snoRNA-associated protein RRP9/U3-55 K along with its role in the development of PC and gemcitabine resistance.

**Methods:**

qRT-PCR, western blot and immunohistochemical staining assays were employed to detect RRP9 expression in human PC tissue samples and cell lines. RRP9-overexpression and siRNA-RRP9 plasmids were constructed to test the effects of RRP9 overexpression and knockdown on cell viability investigated by MTT assay, colony formation, and apoptosis measured by FACS and western blot assays. Immunoprecipitation and immunofluorescence staining were utilized to demonstrate a relationship between RRP9 and IGF2BP1. A subcutaneous xenograft tumor model was elucidated in BALB/c nude mice to examine the RRP9 role in PC in vivo.

**Results:**

Significantly elevated RRP9 expression was observed in PC tissues than normal tissues, which was negatively correlated with patient prognosis. We found that RRP9 promoted gemcitabine resistance in PC in vivo and in vitro. Mechanistically, RRP9 activated AKT signaling pathway through interacting with DNA binding region of IGF2BP1 in PC cells, thereby promoting PC progression, and inducing gemcitabine resistance through a reduction in DNA damage and inhibition of apoptosis. Treatment with a combination of the AKT inhibitor MK-2206 and gemcitabine significantly inhibited tumor proliferation induced by overexpression of RRP9 in vitro and in vivo.

**Conclusions:**

Our data reveal that RRP9 has a critical function to induce gemcitabine chemoresistance in PC through the IGF2BP1/AKT signaling pathway activation, which might be a candidate to sensitize PC cells to gemcitabine.

**Graphical Abstract:**

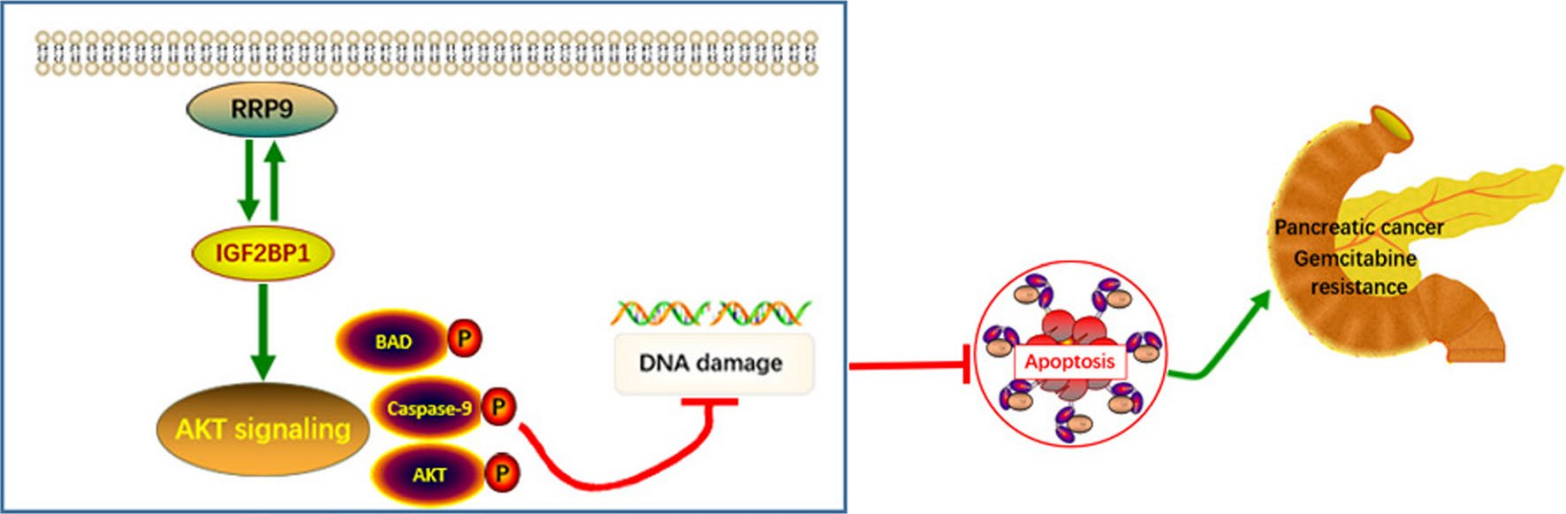

**Video abstract**

**Supplementary Information:**

The online version contains supplementary material available at 10.1186/s12964-022-00974-5.

## Introduction

PC is a common, highly lethal cancer of digestive tract with a survival rate of less than 7% [1]. According to the GLOBOCAN 2020 statistical analysis, PC accounts for 4.7% of cancer-related mortalities worldwide [2]. Treatment strategies include resection surgery, chemotherapy, radiotherapy, targeted therapy and immunotherapy, but are limited due to the fact that by the time a patient presents with PC, the illness is usually at unresectable, advanced stages [3–7]. Furthermore, the development of resistance to traditional chemotherapy and radiotherapy treatment strategies means that therapeutic options such as gemcitabine-based chemotherapy often fail to treat PC [8]. Thus, an increased understanding of molecular mechanisms underlying PC and gemcitabine resistance are required to develop novel treatment strategies, which would improve the therapeutic opportunities currently available to PC patients.

Ribosome biogenesis is a highly complex procedure that occurs in nucleolus and involves the small subunit (SSU)-processome [9]. snoRNAs are predominantly involved in post-transcriptional modification and maturation of ribosomal RNAs (rRNAs), small nuclear RNAs (snRNAs) as well as other cellular RNAs [10]. Since cell growth requires the generation of new ribosomes, it makes sense that cancer cells would exploit the mechanisms involved in ribosome biogenesis to support their accelerated growth rate [11, 12]. Indeed, recent studies have proposed a potential oncogenic role for snoRNAs in various cancers including breast cancer [13, 14], colorectal cancer (CRC) [15, 16], hepatocellular carcinoma (HCC) [17, 18] and pancreatic ductal adenocarcinoma (PDAC) [19].

The RRP9/U3-55 K protein is U3 snoRNA-associated protein that is composed of WD repeat domain [20], which is involved in protein–protein interactions and pre-rRNA processing in the SSU-processome complex [21, 22]. U3 snoRNA is targeted by oncogenes including SIRT7 [23, 24]. The binding of U3 to SIRT7 has been shown to promote the de-acetylation of the U3-55 K component resulting in increased ribosome biogenesis [25, 26]. However, the role of RRP9 in pancreatic cancer drug resistance remains unknown. Thus, RRP9/U3-55 K protein has a potential function in cancer growth and development.

RNA-binding proteins such as insulin-like growth factor 2 mRNA-binding protein 1 (IGF2BP1) have an essential role in embryogenesis and carcinogenesis, and have been implicated as drivers and therapeutic targets in PDAC [27–29]. IGF2BP1 promotes tumor cell proliferation, invasion and chemoresistance through post-transcriptionally regulating its target RNA translation and stability [30]. Recent studies have shown that increased IGF2BP1 expression is a poor prognosis predictor in several tumor types including lung adenocarcinoma [31], HCC [30] and PDAC [32, 33]. Furthermore, in vitro and in vivo experiments have demonstrated that IGF2BP1 could promote PDAC cell proliferation via AKT signaling pathway [32, 33].

Dysregulation of AKT signaling pathway is frequently observed in PC, which is associated with gemcitabine chemoresistance [34]. Thus, identifying and targeting AKT pathway modulators is critical in the development of therapeutic strategies that would reduce gemcitabine resistance. Recently, prominin 2 (PROM2) was found to induce gemcitabine chemoresistance through the AKT signaling pathway in PC [35]. Similarly, IGF2BP1 overexpression in ovarian cancer was illustrated to be associated with cisplatin resistance through AKT phosphorylation [36]. However, IGF2BP1 role in promoting gemcitabine resistance in PC has not been described.

Here, we discovered that RRP9 expression is increased in PC and promotes gemcitabine resistance in vitro and in vivo. Mechanistically, we show that RRP9 activates AKT signaling pathway by interacting with the DNA binding region of IGF2BP1. Treatment of RRP9-overexpressing PC cells with AKT inhibitor MK-2206 and gemcitabine significantly inhibited tumor proliferation. In summary, the data identify RRP9 as a new target, which may prove to be beneficial for PC treatments. Specifically, RRP9 functions indispensably to promote gemcitabine chemoresistance. Thus targeting RRP9 might provide a potential therapeutic strategy to sensitize PC cells to gemcitabine.

## Methods

### Patients and clinical samples

Twenty tumor and paired adjacent normal tissues were obtained from PC patients treated at Ruijin Hospital of Shanghai Jiaotong University of China. For quantitative polymerase chain reaction (qPCR) analysis, specimens were minced and stored in RNAlater (ThermoFisher Scientific, Waltham, MA, USA) for the isolation of total RNA. For western blot and immunohistochemical analyses, protein was isolated from specimens by freezing them in liquid nitrogen or fixing them in 4% paraformaldehyde, respectively. Institutional Review Committee of the Ruijin Hospital of Shanghai Jiaotong University of China approved current investigation in accordance with the guidelines of Helsinki Declaration.

### Survival analysis with Kaplan–Meier plotter web tool

The web-based Kaplan–Meier Plotter (http://kmplot.com/analysis/index.php?p=service) was employed to determine PC patient five-year survival rate. The data on the Kaplan–Meier Plotter website comes from GEO, EGA and TCGA databases. Kaplan–Meier Plotter performed survival analyses based on gene expression levels.

### Cell lines and cell culture

The human pancreatic ductal epithelial cell line (HPDEC1) and PC cell lines (HPDEC1, CFPAC1, HPAC, PanC-1 and BxPC-3) were gained from ATCC. HPAC and PanC-1 cell lines were cultured in DMEM (Invitrogen, Carlsbad, CA, USA) containing 10% fetal bovine serum (HyClone, Logan, UT). CFPAC-1 cells were grown in Iscove’s modified Dulbecco’s medium (Invitrogen). BxPC-3 cells were cultured in RPMI-1640 Medium (Invitrogen). HPDECs were cultured in keratinocyte serum-free medium (Gibco, Grand Island, NY, USA) containing EGF (1 ng/ml) and BPE (50 mg/ml). The cell lines were maintained at 37 °C in humid incubator with 5% CO_2_.

### Vectors, retroviral infection, and transfection

siRNA sequences were designed and chemically synthesized by QIAGEN. The following RRP9 siRNA sequences were used: siRNA#1: sense: 5’- AAUAAGGAGGAUAAGAGUGUC-3’, antisense: 5’- CACUCUUAUCCUCCUUAUUUA-3’; and siRNA#2: sense: 5’- UAAAUAAGGAGGAUAAGAGUG-3’, antisense: 5’- CUCUUAUCCUCCUUAUUUAAG-3’. The following IGF2BP1 siRNA: sense: 5’- AUGUAAAGCUUGUUCAUGGUG-3’, antisense:

5’-CCAUGAACAAGCUUUACAUCG-3’. Control siRNA: sense: 5’.

-UUCUCCGAUCGUGUGACGU-3’, antisense: 5’-ACGUCACACGAUCGGAGAA-3’. Cells were transfected with siRNAs (50 nM) using Lipofectamine 3000 reagent (Invitrogen).

For RRP9 overexpression, cDNA encoding homo sapiens *RRP9* (GenBank accession no. NM_004704.5) was prepared by PCR, sequenced, and separately cloned into pcDNA3.1 lentiviral expression vector (ThermoFisher Scientific). For infection, cells were grown to 70–80% confluence in 12-well plates, which were infected with lentiviral particles and polybrene. GFP-lentiviral particles acted as controls. We collected cells 48 h post-infection and processed for other assays.

### RNA extraction, reverse transcription (RT), and real-time PCR

Total RNA was extracted from PC tissues and cell lines employing TRIzol (Life Technologies, Waltham, MA, USA). We reversed transcribed total mRNA through PrimeScript RT Reagent kit (TaKaRa, Kyoto, Japan). We carried out cDNA amplification and quantification using Bio-Rad CFX qRT-PCR detection system (Applied Biosystems Inc., Foster City, CA, USA) with SYBR Green Master (ROX; Roche, Toronto, ON, Canada). GAPDH was utilized as housekeeping gene, and 2^−ΔΔCt^ method was applied to calculate relative gene expression values.

### Western blotting analysis

We extracted protein from cell and tissue samples by RIPA buffer. Protein quantification was carried out applying BCA Protein Assay kit (Beyotime Institute of Biotechnology, Shanghai, China). We resolved equal quantities of protein through SDS-PAGE, which was transferred to PVDF membranes. After blocking with 5% BSA for two hours, membranes were incubated over the night at 4 °C with following primary antibodies: anti-RRP9 (1: 500, Eterlife), anti-cleaved caspase-3 (1: 500, Abcam), anti-cleaved poly(ADP-ribose) polymerase (PARP) (1: 1000, Abcam), anti-p-AKT (Ser473) (1:2000, Cell Signaling Technology), anti-AKT1 (1:1000, Cell Signaling Technology), anti-p-BAD (Ser136) (1:500, Cell Signaling Technology), anti-BAD (1:1000, Cell Signaling Technology), anti-p-caspase-9 (Ser 196) (1:500, Abcam), anti-caspase-9 (1:1000, Abcam), anti-γ-H2AX (1:5000, Abcam) and anti-IGF2BP1 (1:100, Santa Cruz). Next, we incubated membranes for 1 h at room temperature with horseradish peroxidase-conjugated secondary antibodies (goat anti-rabbit/mouse, PIERCE, Waltham, MA, USA). After stripping, the membrane was re-probed with the loading control antibody, anti-GAPDH (1:1000; Santa Cruz). We visualized protein bands via chemiluminescence that enhanced.

### MTT cell viability assay

PC cell sensitivities to gemcitabine exposure was assessed through MTT assay. Briefly, cells (2 × 10^3^) plated onto 96-well plates were cultured overnight at 37 °C, which were treated by varying gemcitabine concentrations for 1 d. Next, we incubated cells with MTT (0.5 mg/ml, Sigma) for 4 h at 37 °C. We removed culture medium and added DMSO (150 μl) to each well (Sigma). Falcon microplate reader (BD-Labware) was utilized to measure the absorbance at 540 nm.

### Colony formation assay

Briefly, we exposed cells to gemcitabine for 72 h, which were seeded into 24-well plates (8 × 10^2^ cells per plate). Cell cultures were incubated for 10 days at 37 °C in humid incubator with 5% CO_2_. After fixing, we stained colonies employing 0.2% crystal violet, and counted the colony number.

### Apoptosis assay

Apoptosis was examined applying an annexin V-fluorescein isothiocyanate (FITC)/propidium iodide (PI) kit (Abcam). Cells (1 × 10^6^) were plated in 10-cm plates and treated with gemcitabine for 24 h. We harvested cells, washed them with PBS and re-suspended them in binding buffer (100 μL). Our team incubated samples with annexin V-FITC and PI for 15 min in dark, which were analyzed via FACS (Beckman Coulter, Pasadena, CA, USA).

### Immunofluorescence staining

The co-localization of IGF2BP1 and RRP9 was examined by immunofluorescence staining of PanC-1 or BxPC-3 cells that had been transfected with either the control vector or RRP9-OE plasmid. DNA damage was detected by immunofluorescence staining of γ-H2AX. Briefly, we cultured cells (2 × 10^5^) on glass coverslips that placed in 24-well plates, which were exposed to gemcitabine for 24 h. After fixing with 4% formaldehyde for 15 min, we permeabilized samples with 1% Triton X-100 for 20 min, which were washed with PBS, blocked in 5% BSA for 30 min, then incubated with the following primary antibodies: anti-RRP9 (1: 100, Eterlife), anti-IGF2BP1 (1:50, Santa Cruz) or anti-γ-H2AX (1:250, Abcam, Cambridge, MA, USA) at 4 °C over the night. After washing with PBS twice, we incubated samples with Alexa Fluor either 488-labeled anti-rabbit IgG or 594-labeled anti-mouse secondary antibodies (Thermo, Waltham, MA) for 1 h. Our team stained nuclei with DAPI. Samples were visualized by laser scanning confocal microscopy.

### Co-immunoprecipitation assay

Our group lysed cells with RIPA buffer including broad-spectrum protease inhibitors. We incubated protein (1 mg) with 3 µg anti-RRP9 IgG and anti-IGF2BP1 IgG antibodies overnight at 4 °C on a rotator. We added protein A agarose beads (Santa Cruz Biotechnology), and incubated samples for a further 2 h at 4 °C. We washed agarose beads and extracted proteins that immunoprecipitated, which were subjected to western blot analysis. RRP9 and IGF2BP1 combination was predicted through Starbase (http://starbase.sysu.edu.cn/index.php).

### Luciferase assay

Our group cultured cells (1 × 10^4^) in 48-well plates for 1 d. Transfection of control or AKT-luciferase (AKT-luc) reporter plasmids (100 ng) and pRL-TK renilla plasmid (1 ng) was carried out applying Lipofectamine 3000 (Invitrogen). Dual Luciferase Reporter Assay Kit (Promega) was utilized to detect luciferase signals.

### Immunohistochemical staining (IHC)

Human PC tissues, paired adjacent normal tissues and xenografts from nude mice were formalin-fixed and paraffin-embedded. We prepared tissue sections, which we stained using primary antibody against RRP9 (1: 400, Eterlife). The staining intensity was scored as follows: 0 (no staining); 1 (light yellow), 2 (yellow brown), and 3 (brown). Then, a value for the staining index (SI) was obtained by multiplying the positively-stained tumor cell percentages by staining intensity.

### Subcutaneous xenograft tumor model

A subcutaneous tumor model was established by randomly dividing BALB/c nude mice into 4 groups (n = 5/group). We subcutaneously injected mice in the left dorsal flank with PanC-1 cells transfected with either i) PanC-1/Vector, ii) PanC-1/RRP9-OE, iii) PanC-1/siRNA-Vector or iv) PanC-1/RRP9-siRNA#1 (2 × 106 cells/mouse). For rrp9-siRNA mice, after inoculation of cells into mice, mice were injected with RRP9-siRNA#1 every 4 days to maintain efficacy. The mice were administered vehicle (Control) or gemcitabine (100 mg/kg) intraperitoneally twice a week for 41 d. The PanC-1/RRP9-OE group was treated with gemcitabine plus control or gemcitabine plus the AKT inhibitor MK-2206 (120 mg/kg body weight, 3 times/week) for 41 days. The tumor length and width were detected to evaluate tumor growth. Tumor volume was calculated by (L × W2)/2. An IVIS imaging system was used to monitor the tumors. At the end of experiments, we euthanized animals, removed and weighed their tumors. Formalin-fixed paraffin-embedded samples were prepared. The level of apoptosis in the paraffin-embedded tissue sections was determined by TUNEL assay kit (Promega). Institutional Animal Care and Use Committee of Ruijin Hospital affiliated to Shanghai Jiaotong University approved experimental procedures.

### Statistical analysis

We performed statistical analysis using SPSS 11.0 statistical package with following tests: Fisher’s exact test, Chi-square test, log-rank test and Student’s 2-tailed *t* test. Multivariate statistical analysis was carried out with Cox regression model. Data are denoted by mean ± standard deviation (SD). P < 0.05 was considered as statistical significance.

## Results

### RRP9 expression correlates to poor prognosis and lower survival rates of PC patients

**Fig. 1:**
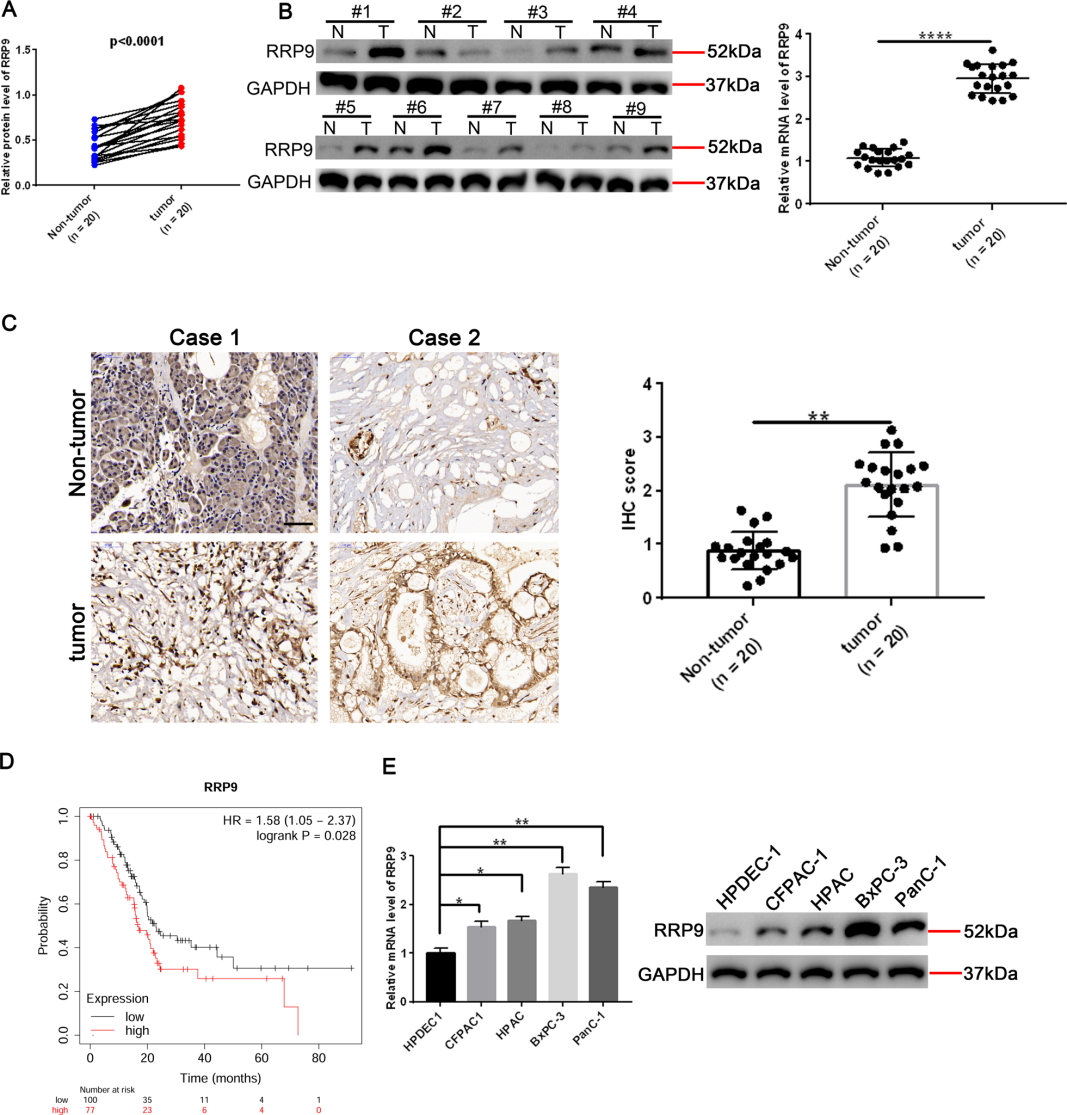
RRP9 expression correlates to poor prognosis and low survival rates in PC patients. **A** RRP9 mRNA levels in 20 paired PC patient samples and adjacent non-tumor tissue. Data are denoted by mean ± SD, n = 20. **B** RRP9 protein levels in 11 paired PC patient samples and adjacent non-tumor tissue. Data are represented by mean ± SD, n = 11. **C** Immunohistochemical staining of RRP9 protein expression in human PC and adjacent non-tumor tissue, (pancreatic non-tumor tissue n = 20, PC n = 20, scale bar: 50 μm). **D** Kaplan–Meier analysis showing overall survival time of PC patients with different RRP9 expression levels, *p* values as indicated. (n = 177, *p* < 0.05). **E** RT-qPCR and western blot detections were carried out to measure RRP9 mRNA (left) and protein (right) expression levels in control HPDEC1 and PC cell lines PanC-1, CFPAC1, HPAC and BxPC-3 cells. Data are expressed by mean ± SD, n = 3, *p < 0.05, **p < 0.01, ****p < 0.0001

RRP9 mRNA and protein levels were significantly higher in human PC samples than normal adjacent tissue (Fig. 1A, B). Similarly, IHC staining revealed elevated RRP9 expression in PC tissue compared to non-tumor tissue (Fig. 1C). Kaplan–Meier analysis informed that the overall PC patient survival rate was lower in patients expressing high levels of RRP9 (Fig. 1D). Significantly elevated RRP9 mRNA and protein levels were also observed in pancreatic cell lines (CFPAC1, HPAC, BxPC-3 and PanC-1) comparing to the control human pancreatic ductal epithelial cell line (HPDEC1) (Fig. 1E). Taken together, these findings demonstrate that elevated RRP9 expression is associated with poor prognosis and lower survival rate of PC patients.

### RRP9 overexpression induces resistance to gemcitabine in PC cells

**Fig. 2:**
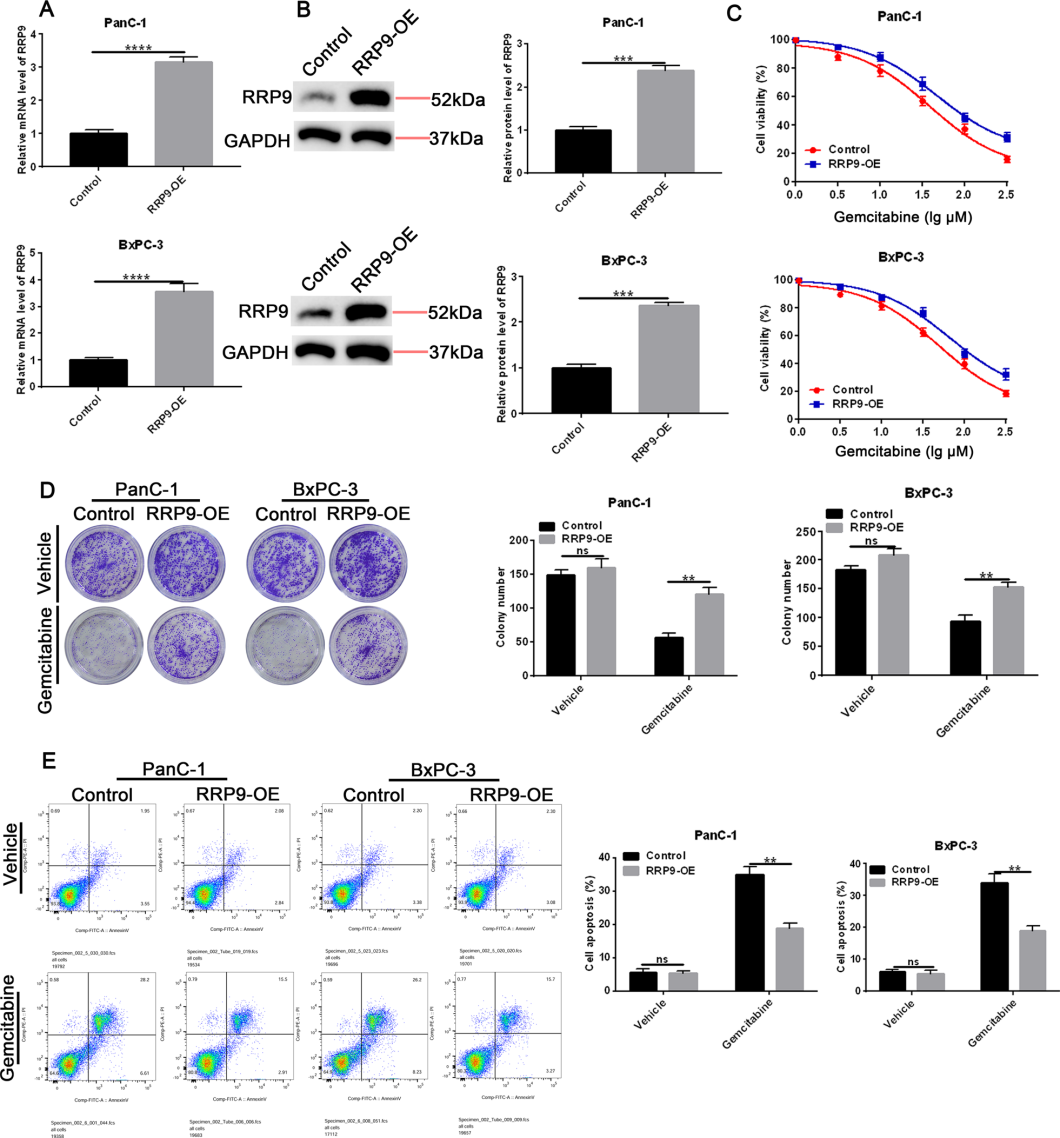
RRP9 overexpression promotes chemoresistance to gemcitabine in PC cells. **A** RRP9 mRNA levels in control cells and RRP-9-overexpressing (RRP9-OE) cells. Data are given as mean ± SD, n = 3. **B** RRP9 protein levels in control cells and RRP-9-overexpressing (RRP9-OE) cells. Data are expressed by mean ± SD, n = 3. **C** Cell viability was assessed in control and RRP9-overexpressing cells treated with increasing concentrations of gemcitabine. Data are given as mean ± SD, n = 50 μM. IC50 of gemcitabine in the indicated cells. **D** Colony formation was measured in control and RRP9-overexpressing cells treated with vehicle or gemcitabine (50 μM). Representative colony formation images (left) and quantification (right) are shown. Data are presented as mean ± SD, n = 3. **E** Apoptosis was measured in control and RRP9-overexpressing cells treated with vehicle or gemcitabine (50 μM). FACS analysis (left) and quantification (right) are given. Data are expressed by mean ± SD, n = 3. Note: **p < 0.01, ***p < 0.001, ****p < 0.0001

To determine whether RRP9 has a function to promote gemcitabine resistance in PC, we transfected BxPC-3 and PanC-1 cell lines utilizing a RRP9 overexpression vector (RRP9-OE). As shown in Fig. 2A-B, treatment with RRP9-OE significantly incremented RRP9 mRNA and protein levels in PanC-1 and BxPC-3 cells. After gemcitabine treatment, cell viability was slightly higher in cells overexpressing RRP9 than control cells (Fig. 2C), while colony formation was significantly higher in RRP9-overexpressing cells than control cells (Fig. 2D). FACS analysis revealed a reduction in apoptosis in RRP9-OE-treated cells after exposure to gemcitabine (Fig. 2E). The effects of gemcitabine on DNA damage in RRP9-overexpressing PC cells were also examined (Additional file 1: Fig. 1). Overexpression of RRP9 resulted in lower levels of DNA damage in gemcitabine-treated cells as measured by decreased expression of the DNA double strand break marker γ-H2AX, and the apoptotic markers, cleaved caspase-3 and cleaved PARP (Additional file 1: Fig. 1A-B). In this manner, our data show that overexpression of RRP9 induces resistance to gemcitabine in PC cells.

### Silencing RRP9 promotes gemcitabine chemosensitivity in PC cells

**Fig. 3:**
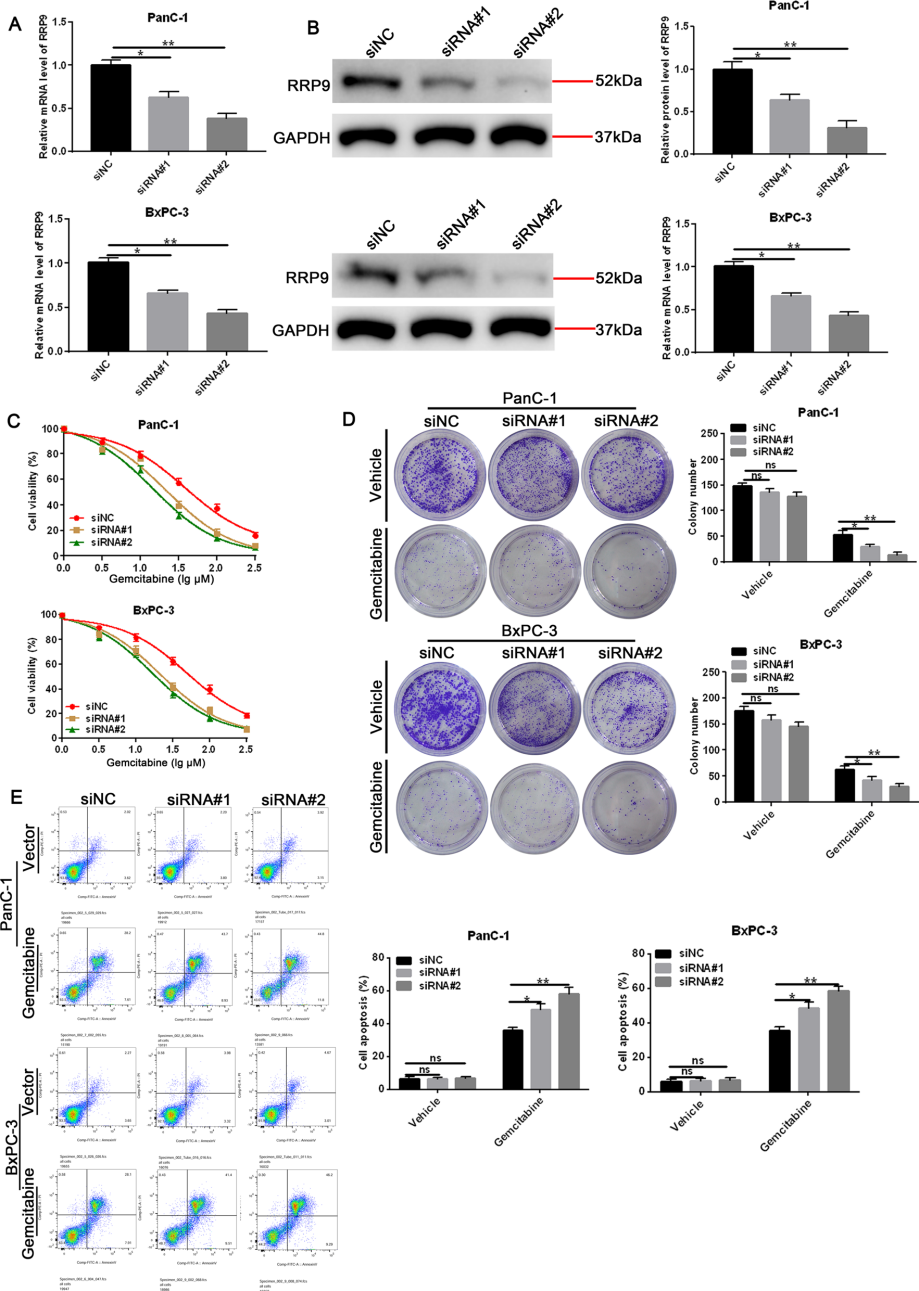
Silencing RRP9 induces gemcitabine chemosensitivity in PC cells. **A** RRP9 mRNA levels in control cells and RRP-9-siRNA-treated (siRNA#1 and siRNA#2) cells. Data are expressed by mean ± SD, n = 3. **B** RRP9 protein levels in control cells and RRP-9-siRNA-treated (siRNA#1 and siRNA#2) cells. Data are denoted by mean ± SD, n = 3. **C** Cell viability was assessed in control and RRP9-silenced cells treated with increasing concentrations of gemcitabine. Data are given as mean ± SD, n = 50 μM. IC50 gemcitabine in cells that indicated. **D** Representative colony formation images (left) and quantification (right) are shown for control and RRP9-silenced cells treated with vehicle or gemcitabine (50 μM). Data are denoted by mean ± SD, n = 3. **E** Apoptosis was measured in control and RRP9-silenced cells treated with vehicle or gemcitabine (50 μM). FACS analysis (left) and quantification (right) of levels of apoptosis. Data are given as mean ± SD, n = 3. Note: *p < 0.05, **p < 0.01

Next, we were to determine the silencing RRP9 effects on gemcitabine-induced chemoresistance in PC cells. As illustrated in Fig. 3A, B, siRRP9 treatment led to a significant reduction in RRP9 mRNA and protein expression levels. Gemcitabine treatment led to decreased cell viability (Fig. 3C) and colony formation (Fig. 3D), together with increased apoptosis (Fig. 3E) in RRP9-silenced PC cells. Furthermore, immunofluorescence staining and western blotting also revealed increased DNA damage and apoptosis as measured by γ-H2AX, cleaved caspase-3 and cleaved PARP expression in RRP9-silenced cells after gemcitabine treatment (Additional file 1: Fig. 1A, B). Our data infer that silencing RRP9 expression promotes gemcitabine sensitivity in pancreatic cells.

### *RRP9 overexpression promotes resistance to gemcitabine in PC *in vivo

**Fig. 4:**
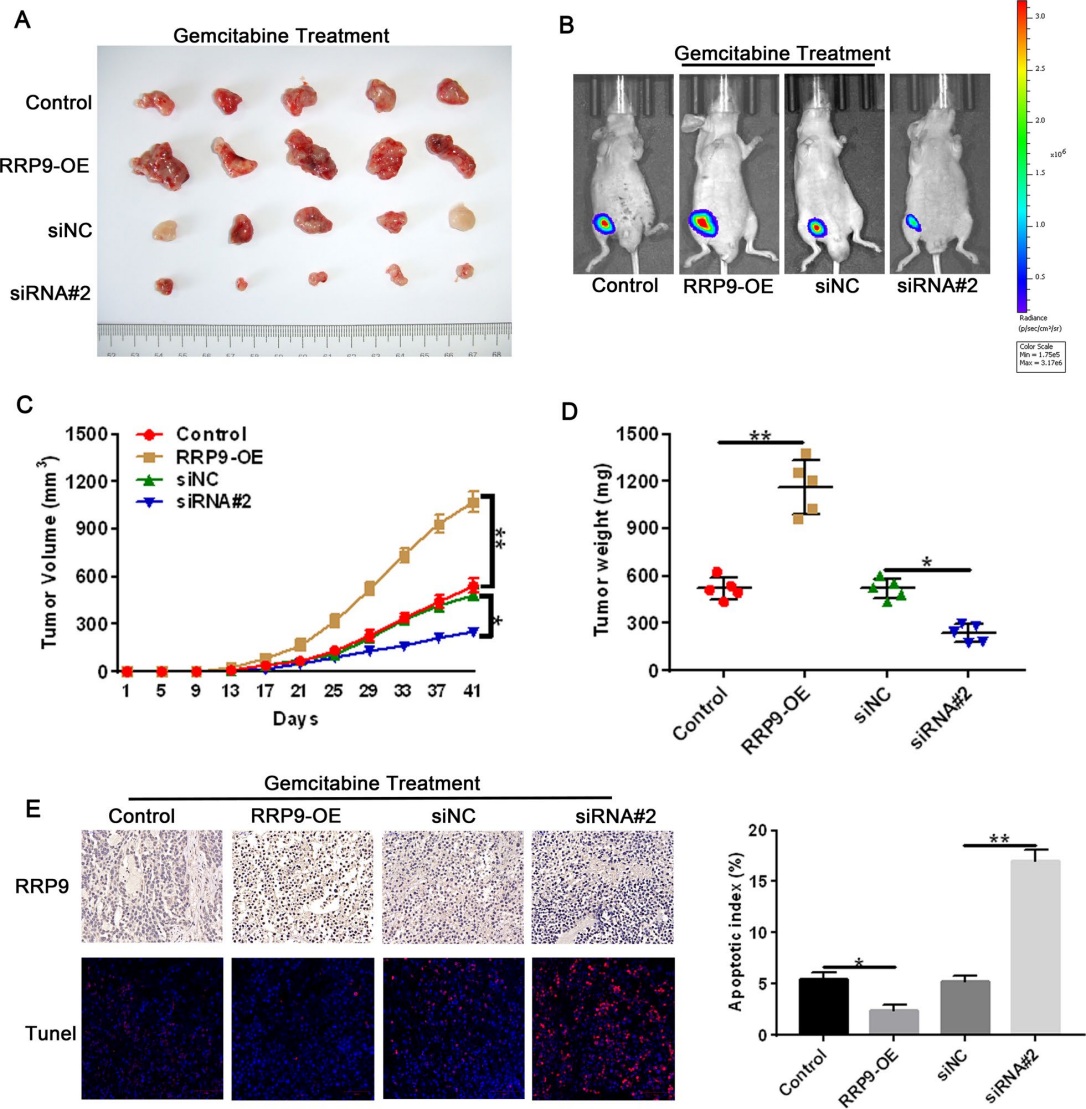
RRP9 overexpression induces gemcitabine resistance in PC in vivo. **A** Representative images showing tumor masses removed from mice with xenografted tumors formed by RRP9-overexpressing or RRP9-silenced cells after gemcitabine treatment. **B** Luminescence signal of xenografted tumors formed by RRP9-overexpressing or RRP9-silenced PC cells in mice after exposure to gemcitabine. **C** Tumor volumes were calculated on timing that indicated. Data are denoted by mean ± SD, n = 5. **D** Tumor weights of the xenografted tumors formed by RRP9-overexpressing or RRP9-silenced cells after gemcitabine treatment. Data are presented as mean ± SD, n = 5. **E** Representative images of RRP9 (scale bar: 50 μm) and TUNEL immunofluorescence staining (scale bar: 100 μm) in xenografted tumors formed by RRP9-overexpressing or RRP9-silenced cells after gemcitabine treatment. Quantification of apoptotic data. Data are denoted by mean ± SD, n = 3. Note: *p < 0.05, **p < 0.01

To determine whether RRP9 has a role in mediating gemcitabine sensitivity in PC cells in vivo, we established a subcutaneous xenograft tumor model. We found that in response to gemcitabine treatment, RRP-9-silenced tumors were significantly smaller than siNC-treated tumors, while tumors overexpressing RRP-9 were significantly larger than control tumors (Fig. 4A, C, D). These observations were confirmed by luminescence signals in the xenografted mice (Fig. 4B). Finally, exposure to gemcitabine caused a significant increase and decrease in apoptotic index of RRP-9-silenced and RRP-9-overexpressing tumors, respectively (Fig. 4E). Taken together, our data show that RRP9 overexpression induces resistance to gemcitabine in PC cells in vivo, which suggest that inhibiting RRP9 may promote gemcitabine sensitivity.

### Overexpression of RRP9 activates the AKT signaling pathway in PC cell lines

**Fig. 5:**
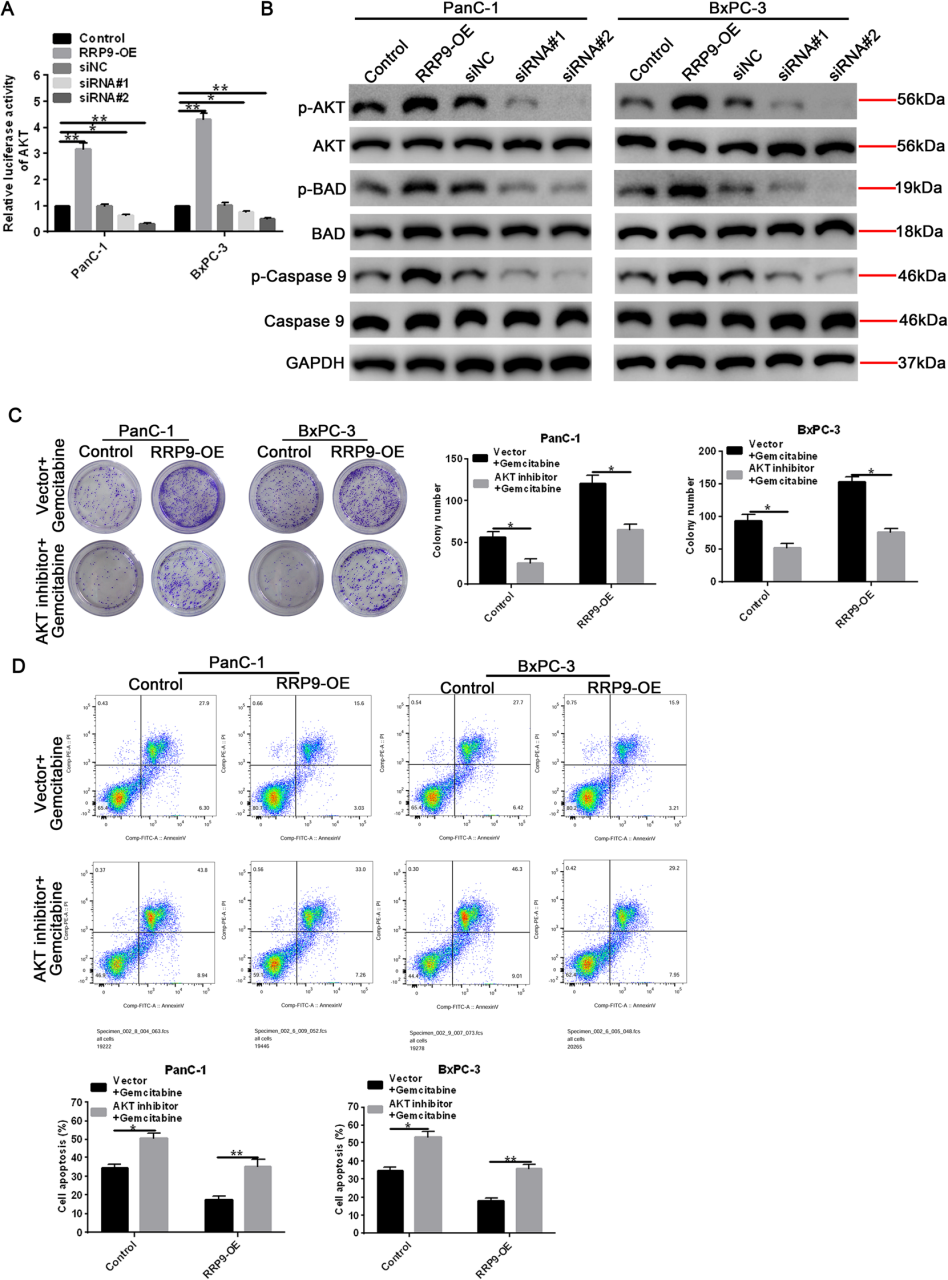
RRP9 overexpression activates the AKT signaling pathway in PC cells. **A** AKT activity was measured in RRP9-overexpressing or RRP9-silenced cells with a luciferase reporter assay. Data are expressed as mean ± SD, n = 3. **B** Western blot analysis of AKT and apoptotic markers, BAD and caspase-9, protein expression in RRP9-overexpressing or RRP9-silenced cells. GAPDH acted as the loading control. **C** Colony formation was measured in control and RRP9-overexpressing cells treated with gemcitabine or gemcitabine plus AKT inhibitor (MK-2206). Representative colony formation images (left) and quantification (right) are shown. Data are presented as mean ± SD, n = 3. **D** FACS analysis (upper panel) and quantification (lower panel) of control and RRP9-overexpressing cells treated with gemcitabine or gemcitabine plus AKT inhibitor. Data are expressed as mean ± SD, n = 3. Note: *p < 0.05, **p < 0.01

Our team sought to determine the mechanism of RRP9 action in PC. Using luciferase assays with the AKT-luc plasmid, we found that overexpression of RRP9 led to significantly increased luciferase activity, indicating that RRP9 activated AKT (Fig. 5A). In contrast, RRP9-silencing resulted in decreased luciferase activity (Fig. 5A). Next, we examined the downstream effects of AKT activation on apoptosis using western blot analysis to assess alternations in apoptotic markers, BAD and caspase-9 (Fig. 5B). We found that overexpression of RRP9 led to increased p-AKT, p-BAD and p-caspase-9 protein levels, whereas silencing RRP9 expression led to decreased expression of p-AKT, p-BAD and p-caspase-9 (Fig. 5B). Next, we examined whether RRP9 promoted gemcitabine resistance via the AKT signaling pathway. Increased colony formation was observed in RRP9-overexpressing cells after gemcitabine treatment. However, after the AKT inhibitor, i.e., MK-2206 treatment, colony formation was significantly decreased (Fig. 5C). FACS analysis revealed that treatment of RRP-overexpressing cells with the AKT inhibitor resulted in significantly higher levels of apoptosis (Fig. 5D). Thus, our findings suggest that RRP9 acts via the AKT signaling pathway to promote PC survival and gemcitabine resistance.

### RRP9 promotes gemcitabine resistance through the IGF2BP1/AKT signaling pathway in PC cell lines

**Fig. 6:**
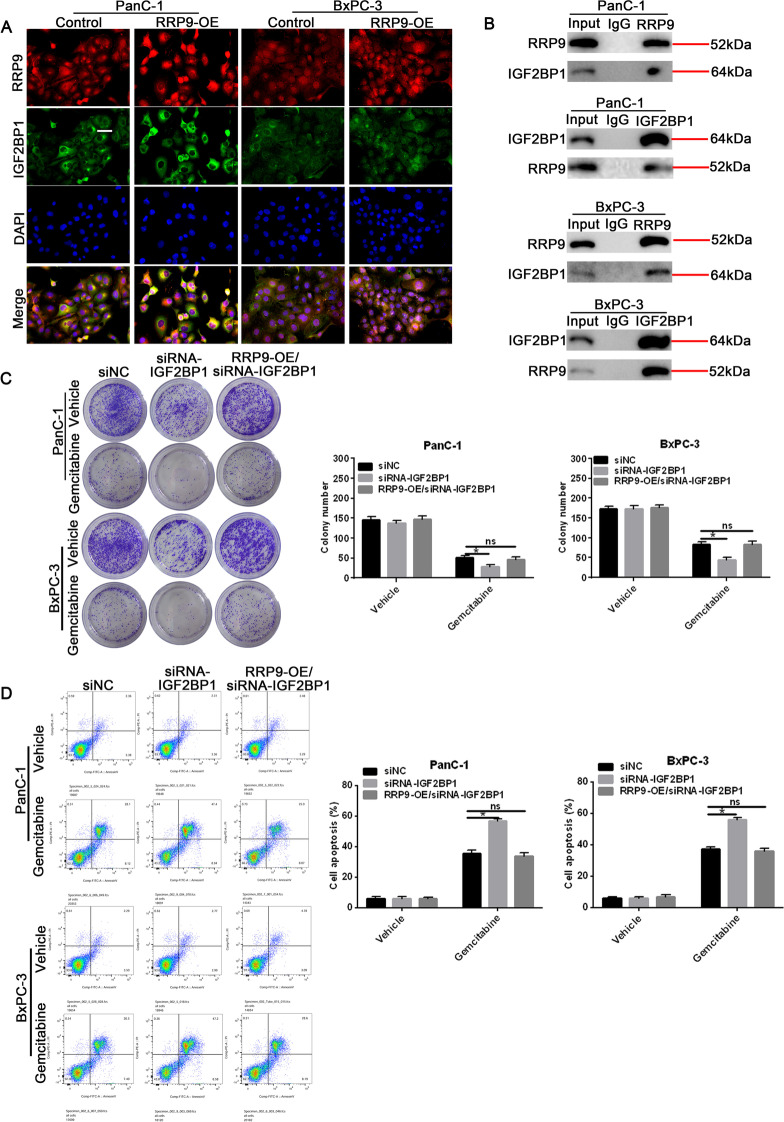
RRP9 induces gemcitabine resistance through the IGF2BP1/AKT signaling pathway activation in PC cell lines. **A** Immunofluorescence staining showing co-localization of IGF2BP1 and RRP9 in control and RRP-overexpressing PC cells. Scale bar, 20 μm. **B** Immunoprecipitation assay demonstrating that RRP9 interacts with IGF2BP1 in control and RRP-overexpressing PC cells. **C** Colony formation was assessed in control, siRNA-IGF2BP1 or RRP9-OE/siRNA-IGF2BP1 cells treated with gemcitabine (50 μM). Representative colony formation images (left) and quantification (right) are shown. Data are presented by mean ± SD, n = 3. **D** FACS analysis (left) and quantification (right) in control, siRNA-IGF2BP1 or RRP9-OE/siRNA-IGF2BP1 cells treated with gemcitabine (50 μM). Data are expressed as mean ± SD, n = 3. Note: *p < 0.05

AKT signaling pathway dysregulation has previously been linked to gemcitabine resistance development in PC [34]. Recently, IGF2BP1 was found to enhance cell proliferation in PC cells through AKT signaling pathway [32]. Thus, we sought to determine whether RRP9 mediated its gemcitabine-resistant effects via the IGF2BP1/AKT pathway. First, we demonstrated that IGF2BP1 co-localized with RRP9 in PanC-1 and BxPC-3 cells (Fig. 6A), which confirmed that IGF2BP1 bound to RRP9 using immunoprecipitation assays (Fig. 6B). Using siRNA-IGF2BP1 to silence IGF2BP1 expression, we examined the effects of RRP9 overexpression and IGF2BP1 silencing on colony formation in vehicle- and gemcitabine-treated PC cells. We found that silencing IGF2BP1 in vehicle-treated cells led to slight reduction in colony formation, while RRP9 overexpression in IGF2BP1-silenced cells led to increased colony formation (Fig. 6C). In contrast, after gemcitabine treatment, knockdown of IGF2BP1 caused significant reduction in colony formation, even in cells that overexpressed RRP9 (Fig. 6C). Consistent with these findings, FACS analysis revealed that silencing IGF2BP1 caused significant increase in apoptosis in gemcitabine-treated cells, which was reduced by overexpression of RRP9 (Fig. 6D). An increase in double strand breaks as measured by γ-H2AX was observed after knockdown of IGF2BP1 in gemcitabine-treated cells (Additional file 2: Fig. 2). Similarly, silencing IGF2BP1 resulted in decreased p-AKT protein expression together with decreased p-BAD and p-caspase-9 in gemcitabine-treated cells (Additional file 3: Fig. 3). Taken together, these findings suggest that RRP9 promotes gemcitabine resistance through IGF2BP1/AKT pathway.

### RRP9 promotes tumor growth and gemcitabine-induced chemoresistance through AKT signaling pathway in PC

**Fig. 7:**
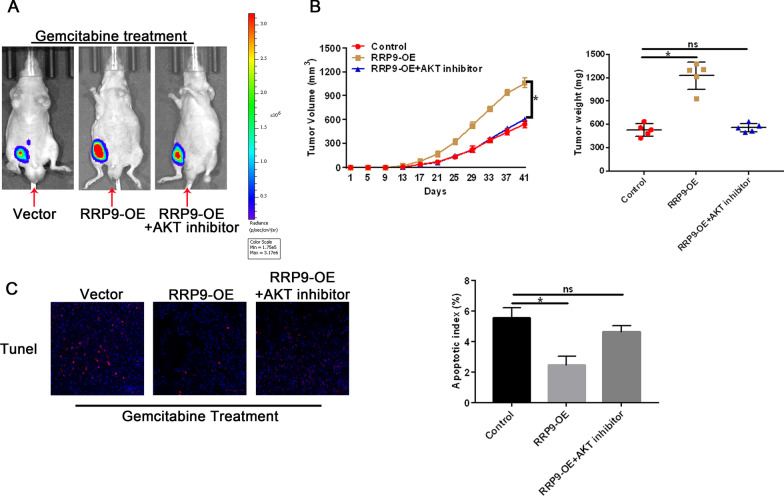
RRP9 promotes tumor growth and chemoresistance to gemcitabine via AKT signaling pathway in PC. **A** Luminescence signal of xenografted tumors formed by vector, RRP9-OE and RRP9-OE + AKT inhibitor-treated cells in mice after gemcitabine treatment. **B** Tumor volumes (left) and tumor weights (right) of xenografted tumors. Data are expressed by mean ± SD, n = X. **C** Representative images showing TUNEL staining of vector-, RRP9-OE- and RRP9-OE + AKT inhibitor-treated tumor sections after gemcitabine treatment (50 μM). Scale bars: 100 μm (left panel). Quantification of percentage of apoptotic cells. Data are given as mean ± SD, n = 3 (right panel). Note: *p < 0.05

Finally, we examined whether RRP9 promoted tumor growth and gemcitabine-induced chemoresistance through AKT signaling pathway in vivo using our mouse subcutaneous xenograft model. We found a significant increase in the tumor volume and weight, as well as luminescence, of RRP9-overexpressing cells after gemcitabine treatment compared to control cells (Fig. 7A, B, C). This response was significantly reduced after inhibition of AKT. TUNEL staining revealed decreased levels of apoptosis in RRP9-overexpressing tumors that were restored back to control levels of levels after inhibition of AKT (Fig. 7C). These findings suggest that RRP9 promotes tumor growth and the development of gemcitabine chemoresistance in mice via the AKT signaling pathway.

## Discussion

Development of effective therapeutic strategies for the PC treatment is challenging because of the PC cell resistance to traditional chemotherapy and radiotherapy options. In the current study, we found that RRP9 induces resistance to gemcitabine in PC in vitro and in vivo. Mechanistically, we confirmed that RRP9 activates the AKT signaling pathway by interacting with the DNA binding region of IGF2BP1. Furthermore, treatment of RRP9-overexpressing pancreatic cells with the AKT inhibitor MK-2206 and gemcitabine significantly inhibited tumor proliferation. Thus, our data illustrate an essential function for RRP9 to promote gemcitabine resistance, and indicate that targeting RRP9 through AKT could propose a new therapeutic strategy to sensitize PC cells to gemcitabine.

snoRNAs, responsible for post-transcriptional modifications of rRNAs during rRNA biogenesis, have been identified as potential biomarkers of various cancers including SNORA71A in HCC [37] and CRC [38], and SNORA23 in PDAC [19]. Here, we found significantly elevated expression of the U3 snoRNA-associated protein, RRP9/U3-55 K, in human pancreatic tissues and PC cell lines. Furthermore, high RRP9 expression correlated with poor patient prognosis and lower survival rates, suggesting that RRP9 could be a potential marker for PC.

The PC cell gemcitabine chemoresistance development means that traditional therapeutic strategies such as chemotherapy are not always an effective treatment option [8]. Thus, recent studies have focused on understanding the mechanisms underlying drug resistance and identifying novel ways to improve drug sensitivity [39–41]. Multiple non-coding RNAs are implicated in gemcitabine resistance in PC including miRNA-3663 [42] and DLEU2L [43]. Here, we discovered that RRP9 overexpression was associated with incremented gemcitabine resistance, while silencing RRP9 expression led to increased sensitivity as shown by decreased colony formation and increased apoptosis in PC cell lines and significantly smaller tumors in our mouse xenograft tumor model. Thus, we identify RRP9 as a candidate target to improve PC sensitivity to gemcitabine.

Aberrant IGF2BP1 expression has been associated with tumorigenesis in different cancers [27, 28]. Although many of the cancer-related mRNA targets of IGF2BP1 were found to promote cell proliferation, migration and invasion [32, 33, 44], several were shown to take part in indirect suppression of tumor growth and metastasis [45, 46]. We found that IGF2BP1 is required for gemcitabine resistance, and that RRP9 interacts with IGF2BP1, leading to reduced apoptosis and increased growth in PC cells. Interestingly, IGF2BP1 is shown to exert is tumorigenic effects in PC through AKT signaling pathway activation [32, 33].

Dysregulation of the AKT pathway has been associated with poor prognosis in PC patients [47]. Furthermore, AKT signaling pathway has been shown to promote gemcitabine chemoresistance through PROM2 [35] and miRNA-93-5p [48]. Here, we found that overexpression of RRP9 also promoted gemcitabine chemoresistance in PC through AKT signaling pathway. Our data discover that targeting AKT signaling pathway may provide a novel strategy to sensitize pancreatic tumors to gemcitabine.

## Conclusions

In conclusion, our data identifies for the first time a role for RRP9 in mediating tumorigenesis and gemcitabine resistance in PC. Critically, inhibition of the IGF2BP1/AKT signaling pathway sensitizes RRP9-overexpressing tumors to gemcitabine. RRP9 promotes tumorigenesis and gemcitabine resistance through the IGF2BP1/AKT signaling pathway. Thus, in addition to previously established methods of sensitizing pancreatic tumor cells to chemotherapeutic agents [49], RRP9 and its downstream effectors may be exploited as novel targets to increase PC chemosensitivity to gemcitabine.

## Supplementary Information

**Additional file 1: Fig. 1.** (A) Representative immunofluorescence images showing DNA damage in control, RRP-9-overexpressing or RRP-9 silenced pancreatic cells after gemcitabine treatment. Green, γ-H2AX; blue, nuclei. Scale bar, 10 μm. (B) Western blot analysis of DNA damage (γ-H2AX) and apoptosis (cleaved caspase-3 and cleaved PARP) markers in control, RRP-9-overexpressing or RRP-9 silenced PC cells after gemcitabine treatment (50 μM). GAPDH acted as the loading control

**Additional file 2: Fig. 2.** (A) Representative immunofluorescence images of DNA damage in control, siRNA-IGF2BP1 or RRP9-OE/siRNA-IGF2BP1 cells after gemcitabine treatment (50 μM). Green, γ-H2AX; blue, nuclei. Scale bar, 20 μm. (B) Western blot analysis of γ-H2AX protein expression in control, siRNA-IGF2BP1 or RRP9-OE/siRNA-IGF2BP1 cells. GAPDH acted as the loading control

**Additional file 3: Fig. 3.** Western blot analysis of protein expression levels regarding p-AKT and the apoptotic markers, BAD and caspase-9, in control, siRNA-IGF2BP1 or RRP9-OE/siRNA-IGF2BP1 cells after gemcitabine treatment (50 μM). GAPDH was used as a loading control

## Data Availability

All data generated or analysed during this study are included in this published article and its supplementary information files.

## Abbreviations

CRC: Colorectal cancer
HCC: Hepatocellular carcinoma
IGF2BP1: Insulin-like growth factor 2 mRNA-binding protein 1
PDAC: Pancreatic ductal adenocarcinoma
PROM2: Prominin 2
rRNA: Ribosomal RNA
snRNA: Small nuclear RNA
snoRNAs: Small nucleolar RNAs
SSU: Small subunit
