# Supplementary figures and images for: RRP9 promotes gemcitabine resistance in pancreatic cancer via activating AKT signaling pathway
Source: Cell Commun Signal. 2022 Nov 24;20:188. doi: 10.1186/s12964-022-00974-5 (PMC9700947; doi:10.1186/s12964-022-00974-5)

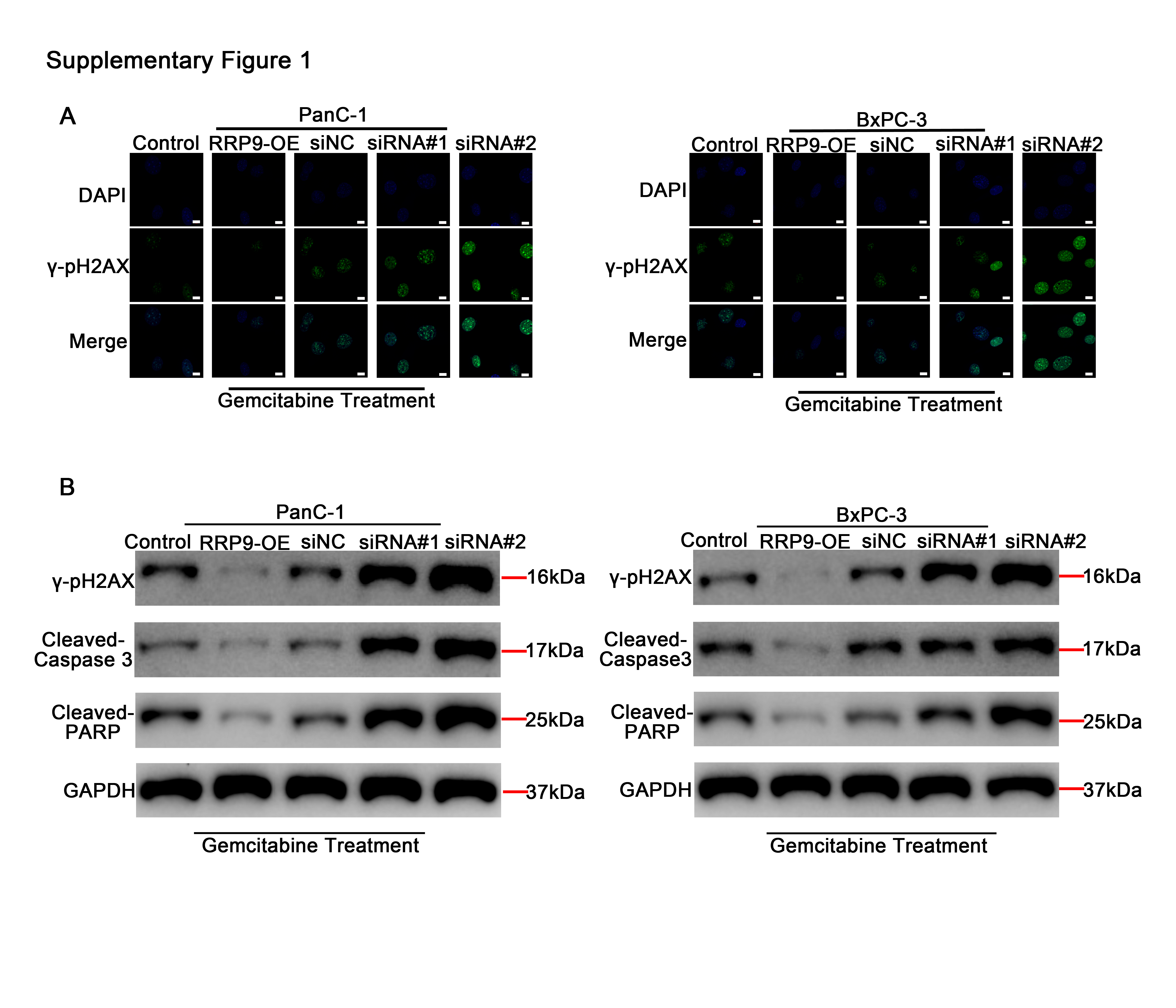

Supplement: Supplementary file 2 — Additional file 1: Fig. 1. (A) Representative immunofluorescence images showing DNA damage in control, RRP-9-overexpressing or RRP-9 silenced pancreatic cells after gemcitabine treatment. Green, γ-H2AX; blue, nuclei. Scale bar, 10 μm. (B) Western blot analysis of DNA damage (γ-H2AX) and apoptosis (cleaved caspase-3 and cleaved PARP) markers in control, RRP-9-overexpressing or RRP-9 silenced PC cells after gemcitabine treatment (50 μM). GAPDH acted as the loading control [file 12964_2022_974_MOESM2_ESM.tif]

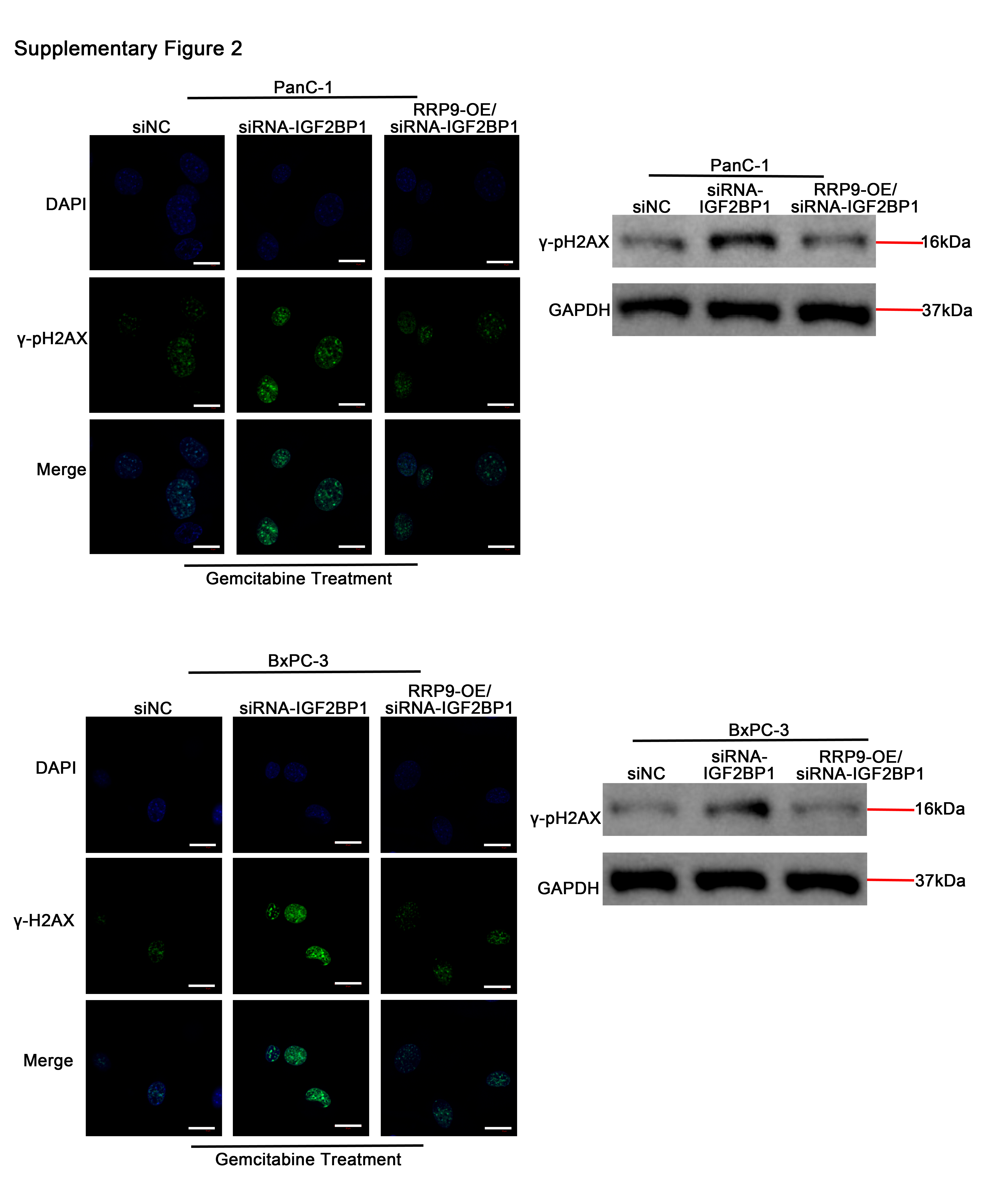

Supplement: Supplementary file 3 — Additional file 2: Fig. 2. (A) Representative immunofluorescence images of DNA damage in control, siRNA-IGF2BP1 or RRP9-OE/siRNA-IGF2BP1 cells after gemcitabine treatment (50 μM). Green, γ-H2AX; blue, nuclei. Scale bar, 20 μm. (B) Western blot analysis of γ-H2AX protein expression in control, siRNA-IGF2BP1 or RRP9-OE/siRNA-IGF2BP1 cells. GAPDH acted as the loading control [file 12964_2022_974_MOESM3_ESM.tif]

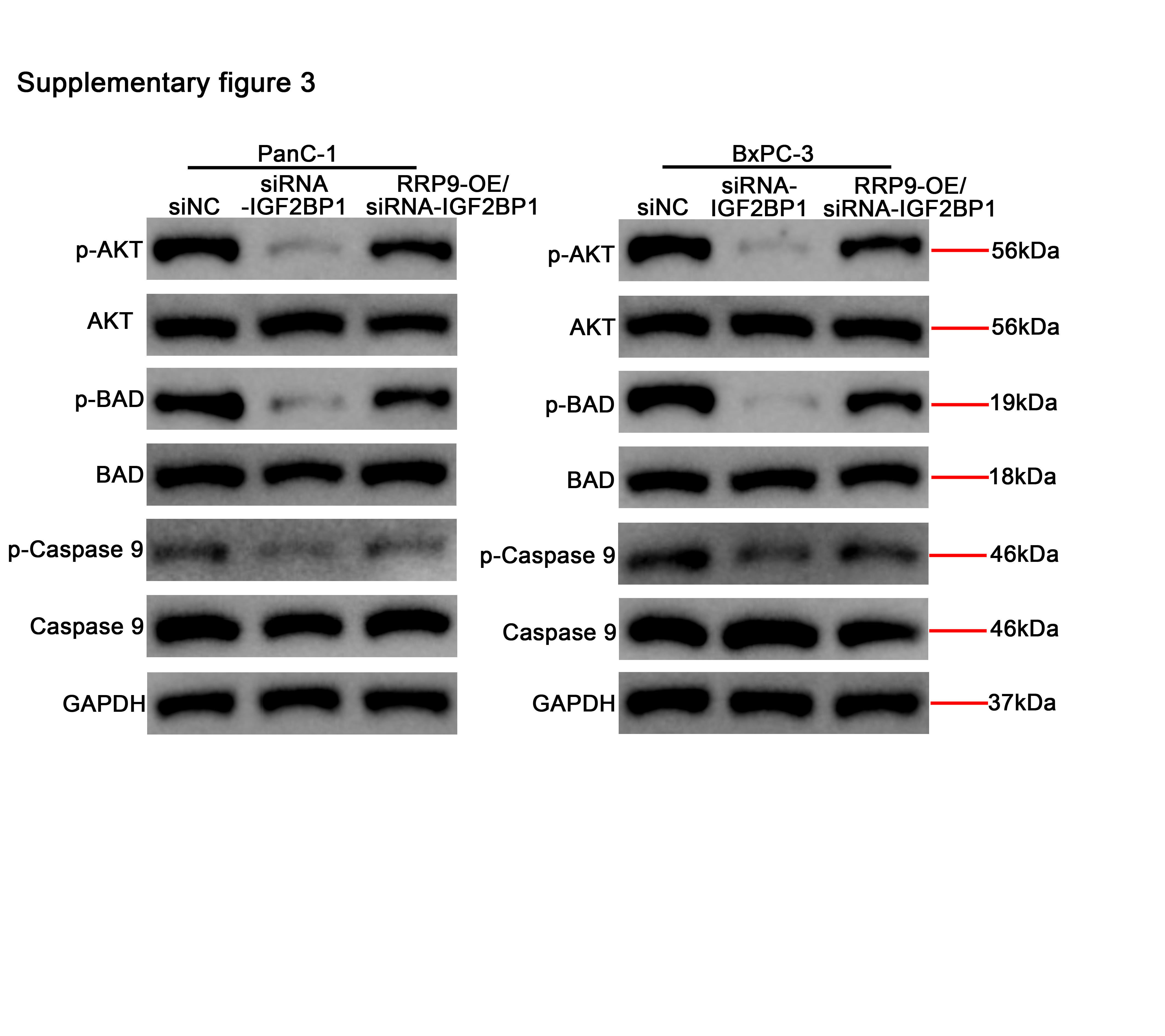

Supplement: Supplementary file 4 — Additional file 3: Fig. 3. Western blot analysis of protein expression levels regarding p-AKT and the apoptotic markers, BAD and caspase-9, in control, siRNA-IGF2BP1 or RRP9-OE/siRNA-IGF2BP1 cells after gemcitabine treatment (50 μM). GAPDH was used as a loading control [file 12964_2022_974_MOESM4_ESM.tif]
